# Topical Bacteriophage Therapy for Staphylococcal Superficial Pyoderma in Horses: A Double-Blind, Placebo-Controlled Pilot Study

**DOI:** 10.3390/pathogens12060828

**Published:** 2023-06-14

**Authors:** Kalie Marshall, Rosanna Marsella

**Affiliations:** Department of Small Animal Clinical Sciences, College of Veterinary Medicine, University of Florida, 2015 SW 16th Avenue, Gainesville, FL 32610, USA; kaliemarshall@ufl.edu

**Keywords:** pyoderma, staphylococcus, bacteriophage, horse

## Abstract

Increased antimicrobial resistance highlights the need for alternatives to antibiotics. Bacteriophages, which are benign viruses that kill bacteria, are promising. We studied the efficacy of topical bacteriophages for treating equine staphylococcal superficial pyodermas. Eight *Staphylococcus aureus* isolates were tested against a bacteriophage bank, and a cocktail consisting of two bacteriophages was prepared. Twenty horses with clinical and cytological evidence of superficial pyoderma and confirmed *S. aureus* infection based on swabbed culture were enrolled in the study. Each horse received both the bacteriophage cocktail and the placebo at two different infection sites, once daily for four weeks. Clinical lesions and cytology were evaluated weekly by an investigator who was unaware of the treatment sites. All infection sites were swabbed and cultured at the end of the study. A linear mixed model showed no significant differences between the placebo and treatment sites in terms of clinical signs, cytological scores of inflammation, and bacterial counts at the end of the study. It is possible that the bacteriophage cocktail killed *S. aureus*, but cytology scores did not change as new populations of cocci took over. The study limitations included a small sample size and inconsistent control of the underlying causes of pyodermas.

## 1. Introduction

With the increased prevalence of antimicrobial resistance (AMR) seen in both human and veterinary medicine, it is extremely important to identify and develop novel treatment options to better equip clinicians in the fight against bacterial infections. Bacteriophages are one of the most promising avenues for research in this aspect [1].

Bacteriophages, also known as phages, are viruses that specifically infect bacteria and use them as their host to multiply. In general, bacterial metabolism is hijacked to amplify viral DNA and to produce bacteriophage proteins. More specifically, once a bacteriophage attaches to a susceptible host, it follows one of two replication strategies, namely lytic or lysogenic replication [2].

During a lytic replication cycle, a phage attaches to a host bacterium, introduces its genome into the host cell cytoplasm, and utilizes the ribosomes of the host to make its proteins. The phage DNA remains separate from the host DNA. The host cell resources are converted to viral genomes and capsid proteins, which assemble into multiple copies of the original phage. As the host cell dies, it is lysed, thereby releasing new bacteriophages to infect other host cells [2]. One of the important enzymes that lytic bacteriophages use to lyse their hosts is endolysin. Endolysins are used at the end of the replication cycle to degrade peptidoglycans of the bacterial host from within, resulting in cell lysis and release of progeny virions [3,4]. A recent study on canine pyoderma found that endolysin of staphylococcal bacteriophage had broader lytic activity against staphylococcal isolates than the staphylococcal bacteriophage itself [5]. 

During a lysogenic replication cycle, a phage also attaches to a host bacterium and introduces its genome into the host cell cytoplasm. However, the phage genome is integrated into the bacterial cell chromosome or maintained as an episomal element, whereby, in both cases, it is replicated and passed onto daughter bacterial cells without killing them. Integrated phage genomes are called prophages, and bacteria containing them are lysogens. Prophages are passed onto daughter cells each time a cell divides. Under stressful conditions, prophages can convert back to a lytic replication cycle where the phage DNA is excised from the bacterial chromosome and kills their host. This most often occurs in response to changing environmental conditions [2]. For the purpose of clinical treatment, it is important to have lytic bacteriophages that kill the host bacterium.

Because bacteriophages closely follow the course of bacterial growth, they naturally evolve alongside bacteria to infect new strains as they diverge and circumvent bacterial resistance to infection as such resistance arises. With an estimation of 10^31^ bacteriophages on earth, the notion of bacterial resistance to them appears unattainable [6,7].

The process of identifying and developing bacteriophages is far quicker and easier than the process of developing new antibiotics. Moreover, bacteriophages can be combined to attack a wide range of bacterial strains and help prevent the development of resistance. Previous studies have indicated that a bacteriophage cocktail (>2 phages) can delay the appearance of bacteriophage-resistant bacterial variants and enhance treatment efficacy [8,9]. Such a cocktail can target a single species or a broad range of pathogenic bacteria and provide a greater potential for presumptive or empirical treatment relative to individual bacteriophage isolates [10].

Bacteriophages offer several advantages over common antibiotics, including less safety concerns; minimal collateral damage to the human host’s healthy microbiome; bactericidal efficacy regardless of AMR profiles; potential synergy with antibiotics; potential reversion of susceptibility to antibiotics; activity against bacterial biofilms; and anticipated cost-effectiveness of pharmaceutical development [11,12,13,14,15,16,17,18]. Biofilm-related infections have caused increased concern regarding tolerance to antibiotics in recent years. Therefore, bacteriophage activity against biofilms is of great importance. Bacteriophages have been shown to be more efficient in biofilm biomass removal and in inducing a reduction in staphylococcus count (a common producer of biofilms) compared to antibiotics [19].

Bacteriophage therapy fell out of favor in the Western world with the advent of antibiotics [20]. When penicillin was discovered, the concept of individual phages attacking one bacterium at a time was deemed less useful. However, its use continued in some former Soviet countries and parts of Eastern Europe. For instance, in the Republic of Georgia, the use of bacteriophage therapy for managing many types of non-systemic infections is an accepted practice [10]. With increased AMR throughout the world, the West has started to take interest in bacteriophage therapy once again [19,20]. In addition, their specificity, which has once made bacteriophages seem less desirable, is now their greatest appeal in regard to AMR bacteria. 

A limited number of controlled human clinical trials have been conducted with bacteriophages and that number is even smaller in veterinary medicine. The first published clinical trial in veterinary medicine focused on the efficacy of a phage cocktail against *Pseudomonas aeruginosa* otitis externa in dogs [21]. Treatment using the phage cocktail resulted in a 30.1% reduction in clinical scores in 10 dogs after 48 h [21]. Additionally, there was a 67% reduction in *P. aeruginosa* counts after 48 h, and a parallel increase in phage titers, with up to a 100-fold increase in four dogs treated with the phage cocktail [21]. Another veterinary medicine disease that has been studied using bacteriophages is equine bacterial keratitis. A mouse model was used to show that phages could prevent keratitis caused by *Pseudomonas aeruginosa* [22]. Veterinary studies of superficial pyoderma treated with a topical bacteriophage cocktail, like the one proposed in this study, have yet to be published as, until recently, all the phages discovered against *S. pseudintermedius*, the staphylococcus responsible for most canine pyodermas, are lysogenic and not lytic [4]. It is only recently that a lytic phage for *S. pseudintermedius* has been reported [23]. By identifying the sources and strains of staphylococcal infection and developing an effective bacteriophage cocktail to treat such infection, we could enable effective, low-cost treatment and prevention of AMR infections.

Overall, staphylococcal skin infections are common in veterinary dermatology and are frequently treated with antibiotics prior to the development or identification of AMR. Human studies in Poland have shown a 90% success rate with bacteriophage therapy against cases of *Staphylococcus aureus* [24]. This shows promise for equine medicine as the three most clinically important staphylococci in horses are *S. aureus*, *S. intermedius*, and *S. hyicus*. Of these three bacterial strains, *S. aureus* has been reportedly isolated most frequently in horses with skin lesions [25]. Additionally, safety and efficacy studies using phage cocktails containing *S. aureus*-specific bacteriophages indicated that there were no adverse effects when administered topically and they were effective in controlling *S. aureus* infection in humans [26,27,28]. 

Equine superficial bacterial pyoderma can be challenging to treat. Skin infections require long periods of treatment due to the nature of the infection; most systemic antibiotics do not reach the skin well, requiring doses higher than those used for infections in other organs, as well as longer treatment times. These factors place patients at risk for adverse effects, especially horses. Horses are hindgut fermenters that rely on normal microflora to digest fiber, which is the main food source in the equine diet. With high doses of antimicrobials for long periods, this normal microflora can be altered and cause profound changes, such as diarrhea and colic. Due to these side effects, veterinarians are limited to the options of antibiotics that can be safely used in horses. Therefore, with increased AMR in horses, there are even fewer options to choose from. Besides the increased side effects seen with systemic antibiotics, they can also be very costly for horses of a large size that need them for long periods. This is a serious limitation for many horse owners. Additional difficulties with horses are their outdoor lifestyle, thicker hair coats, and large body mass. Outdoor environments can be dirty and moist. Increased moisture on the skin can harbor infection and lead to a vicious cycle of reinfection while an infection is being treated. Thick hair coats and large body mass make it difficult for owners to treat horses topically with shampoos that need to be lathered on and require adequate contact time on the horses prior to rinsing. Bacteriophages decrease the labor intensiveness of this by only needing to be applied without needing to be lathered or rinsed. 

Devising a topical formulation of bacteriophages that can effectively combat staphylococcal infections has the potential to reduce the use of antibiotics and, therefore, eventually reduce AMR in animals. The hypothesis tested in this proof-of-concept study was that a topical bacteriophage cocktail would be more effective than placebo in decreasing clinical and cytological signs of pyoderma in horses. Following this study, larger studies can be completed to better assess the clinical application of bacteriophages in equine pyoderma. 

## 2. Materials and Methods

### 2.1. Bacteriophage Screening and Topical Preparation

Staphylococcal strains used for the propagation and counting of bacteriophages consisted of isolates obtained from horses owned by the author’s institution and privately-owned horses in the local area. Bacterial strains were identified using a commercial matrix-assisted laser desorption ionization–time of flight mass spectrometry (MALDI-TOF MS) platform. 

Eight pathogenic *Staphylococcus aureus* isolates from horses at the author’s institution and from privately owned horses in the area were screened against Adaptive Phage Therapeutic’s (APT, Gaithersburg, MD, USA) PhageBank™ using their in vitro Phage Susceptibility Test. The plates for phage susceptibility testing were prepared as described previously [29]. Briefly, bacterial colonies were sampled after overnight incubation and incubated in tryptic soy broth (TSB) at 37 °C until they were visually turbid and then diluted in cold TSB and placed on ice. The assay media were prepared by diluting 100× Biolog tetrazolium (Tz). Phage and bacteria were aliquoted into 96-well assay microplates (Biolog), as previously described, and the plates were loaded onto an OmniLog^®^ instrument (Biolog) for 48 h incubation at 37 °C, with colorimetric reads taken every 15 min.

Two lytic bacteriophages with 100% in vitro inhibition coverage against all eight *S. aureus* strains screened were selected for the topical preparation. The phages tested in our study both belonged to the *Podoviridae* cluster D, which have been previously characterized as lytic [30]. To confirm their lytic properties, APT conducted both phenotypic and genomic investigations.

Once the desired bacteriophages were selected, they underwent amplification, purification, formulation, and quality control at APT. The bacteriophage preparation was stored frozen at −80 °C and contained a concentration of ≥7 × 10^7^ plaque-forming units (PFU)/mL of each of the therapeutic bacteriophages at 50% each in Plasmalyte A + 20% (*v*/*v*) glycerol. A higher concentration of phages was chosen for this study based on successful intranasal topical solutions as we did not have information on topical application on the skin of animals [31,32]. A placebo was formulated using Glycerol USP (Thermo Fisher Scientific, part number G31-1) and Plasmalyte A injection with a pH of 7.4 (Baxter Healthcare Corporation, Deerfield, IL, USA; part number 2B2544X). The placebo was made in bulk by mixing 800 mL of Plasmalyte and 200 mL of glycerol. Plasmalyte was dispensed first, followed by the addition of glycerol. While adding glycerol, the pipette with the solution was rinsed 8–10 times to rinse off residual glycerol from the pipette. Following the bulk preparation of both the placebo and bacteriophage cocktail, individual aliquots were made with 2.5 mL of the bacteriophage cocktail being added to 5.0 mL Eppendorf Tubes^®^ with a screw cap and 2.5 mL of the placebo added to 5.0 mL Eppendorf Tubes^®^ with a snap cap. The principal investigator was blinded to this process.

### 2.2. Animal Use

This study was approved by the University of Florida’s Institutional Animal Care and Use Committee (IACUC #202011182) and the Veterinary Hospitals Research Review Committee. Informed owner consent was obtained prior to the enrollment of each horse. 

A power analysis using the GPower software ascertained the power of effect. Using 20 horses with an alpha error probability of 0.05, a power of 0.8, and two tails, the effect size was considered medium at 0.66. 

Although there were no anticipated adverse reactions to the topical bacteriophage therapy, if a horse developed irritation, redness, swelling, or discharge due to the treatment, it was stopped immediately, and the horse was withdrawn from the study and treated in accordance with the standard of care. The owners also had the right to withdraw their horse at any time for any reason. 

### 2.3. Clinical Trial

This was a prospective, randomized, placebo-controlled study using privately-owned horses with naturally occurring superficial pyoderma caused by *S. aureus*. Twenty horses with clinical and cytological evidence compatible with staphylococcal pyoderma, including papular eruptions, crusting, scaling, and alopecia along with cocci, were included. There were no age, breed, or sex requirements. Horses were excluded if they had no clinical signs of pyoderma and no cocci on cytology. The withdrawal time for prior systemic or topical antibiotic therapy was a week before bacteriophage administration. Horses with known comorbidities were allowed to stay on systemic therapies as long as no changes had been made in the 4 weeks prior to bacteriophage application.

The selected horses were swabbed for culture prior to enrollment to ensure growth of *S. aureus*. Horses with evident growth were enrolled and were given seven placebo tubes with a blue label and seven bacteriophage tubes with a white label on day 0, on dry ice. The association of the label colors for the placebo and bacteriophage treatment were not known to the principal investigator or horse owners. The owners were instructed to keep the tubes in the dry ice and frozen until treatment. For treatment, one bacteriophage tube and one placebo kept on dry ice were removed and allowed to thaw to room temperature. The owners were instructed to apply one tube to one chosen location and the other tube to a separate chosen location every 24 h for four weeks. The owners were given a diagram on day 0 indicating the locations they would be treating with each tube. The owners were allowed to use fly sprays as they were prior to enrollment in this study, just ensuring that the sprayed areas were dry prior to the application of the placebo or bacteriophage cocktail. No other topical therapy was permitted during the trial. The horses were permitted to wear fly sheets, masks, and boots during the trial.

On days 0, 7, 14, 21, and 28, clinical lesions were scored by the same investigator and the horse owner. Clinical lesions were scored from 0 to 3 (0 being absent and 3 being severe). The evaluated clinical lesions by the investigator included papules/pustules, crusts/collarettes, and alopecia. The body areas evaluated included the two chosen treatment sites as well as the head/ears, neck, dorsum, cranial/caudal ventrum, and front and hind legs. The total score was the sum of the scores of each body region (Table 1). The owners were also asked to score the severity of the dermatitis on a scale of 0–3 (Table 2).

On the same days, cytology of affected areas was performed to monitor infection and inflammatory infiltrate (Table 3). Lastly, culture swabs were collected for bacterial identification prior to enrollment to ensure the growth of *S. aureus* and at the end of the study on day 28. Photographs were taken to objectively document progress. At the end of the study, a global assessment score from 0 to 3 was given to each horse (0 = worse than before; 1 = improved but less than 50%; 2 = improved more than 50%; and 3 = complete resolution). This score was calculated by both the owner and the investigator.

On days 0, 7, 14, 21, and 28, clinical lesions were scored by the investigator. The listed sites and each blinded treatment site were scored.

On days 0, 7, 14, 21, and 28, clinical lesions were scored by the owner. Each blinded treatment site was scored as follows: (1) mild = ≤25% of region affected; (2) moderate = 26–74% of region affected; and (3) severe = 75–100% of region affected.

On days 0, 7, 14, 21, and 28, both treatment sites had cytology performed by the investigator, and inflammatory cell and bacterial counts were recorded. 

### 2.4. Statistics

A linear mixed model was used to test the difference at site 1 versus site 2 between time points, and if the lesion at the sites changed over time. Day was considered a fixed effect, horse was considered a random effect, and an autoregressive type 1 correlation structure was included to account for repeated measures on the same horse over time.

## 3. Results

Prior to the start of the clinical trial, sixteen bacteriophages against *S. aureus* were screened against eight *S. aureus* isolates from the horses. Two bacteriophages in combination had 100% in vitro inhibition coverage against all eight *S. aureus* strains (Table 4). These two bacteriophages were combined to make a bacteriophage cocktail used in the clinical trial. 

A total of 117 horses with clinical and cytological evidence compatible with staphylococcal pyoderma were initially swabbed for culture. *Staphylococcus aureus* was the most prevalent sample isolated (32/117; 27.4%), as expected. The other isolates were identified as follows: *S. delphini* (22/117; 18.8%), *S. hyicus* (16/117; 13.7%), *S. xylosus* (10/117; 8.5%), *S. sciuri* (10/117; 8.5%), *S. intermedius* (8/117; 6.8%), *S. arlettae* (8/117; 6.8%), *S. chromogenes* (7/117; 6.0%), *S. schleferi* (2/117; 1.7%), *S. succinus* (2/117; 1.7%), *S. haemolyticus* (2/117; 1.7%), *S. gallinarum* (2/117; 1.7%), *S. warneri* (1/117; 0.9%), and *S. epidermidis* (1/117; 0.9%). In addition, 11/117 samples were identified as staphylococci without a known speciation, and 12/117 samples did not have any staphylococcus growth. 

**Figure 1:**
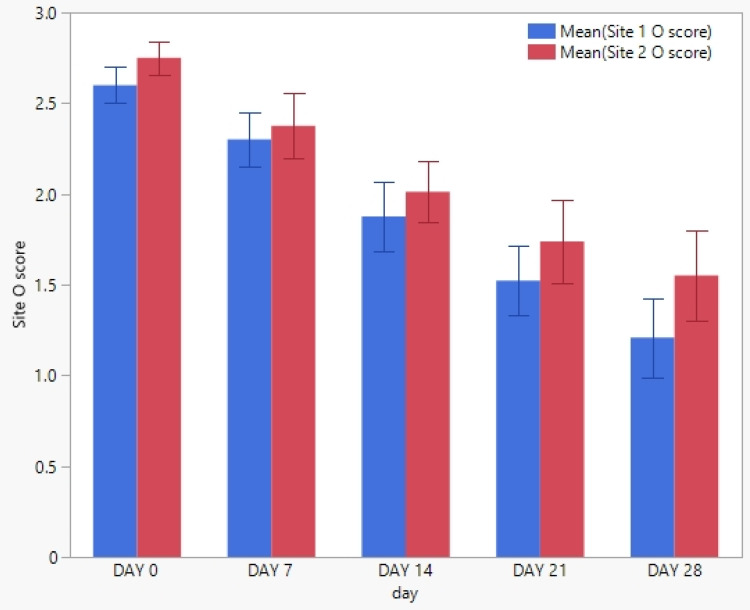
The mean clinical lesion scores and standard deviation from each of the 20 enrolled horses by the owner (O) at each weekly visit. The blue column represents site 1, while the red column represents site 2.

**Figure 2:**
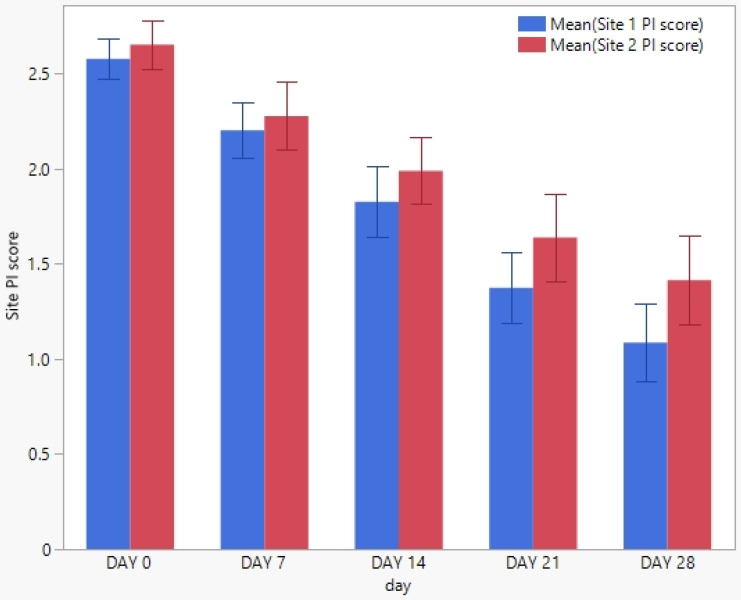
The mean clinical lesion scores and standard deviation from each of the 20 enrolled horses by the principal investigator (PI) at each weekly visit. The blue column represents site 1, while the red column represents site 2.

**Figure 3:**
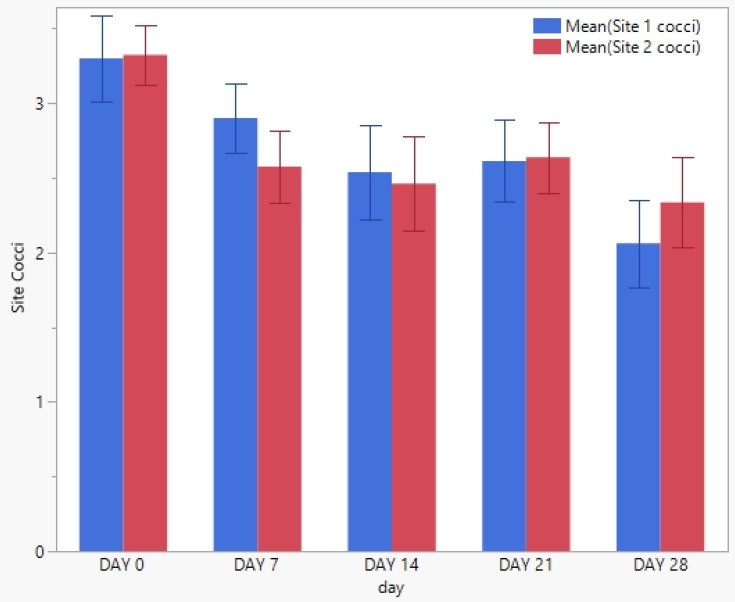
The mean cytological bacterial counts and standard deviation from each of the 20 enrolled horses at each weekly visit. The blue column represents site 1, while the red column represents site 2.

**Figure 4:**
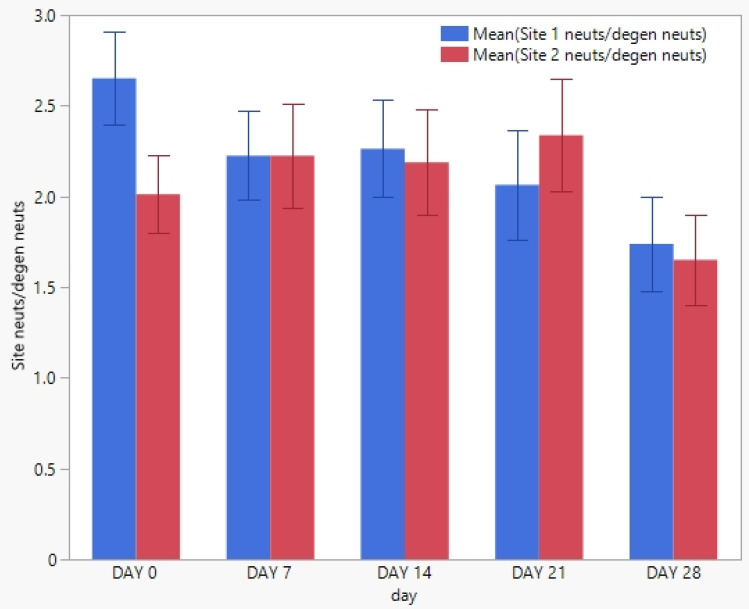
The mean cytological inflammatory cell counts and standard deviation from each of the 20 enrolled horses at each weekly visit. The blue column represents site 1, while the red column represents site 2.

Twenty horses with *Staphylococcus aureus* pyoderma were enrolled in this study. None of the horses enrolled were withdrawn from the study. No significant difference (*p* < 0.05) in the clinical scores from the principal investigator or the owner between any two time points was found (Figure 1 and Figure 2). No significant difference (*p* < 0.05) in cytological counts of cocci or neutrophils between any two time points was found (Figure 3 and Figure 4). 

## 4. Discussion

In our study, the bacteriophage cocktail we used did not improve clinical signs of pyoderma. From the figures above, it can be seen that the clinical scores from the principal investigator and the owner from day 0 to day 28 trend downward, and this is the case for both the placebo and treatment sites. Based on a clinical assessment of many of the horses that were enrolled, it is suspected that most of them likely had an insect bite hypersensitivity as the underlying reason for their pyoderma. Many of the owners required education on insect bite hypersensitivity. They were instructed to increase fly spray regimens with appropriate concentrations of active ingredients, as well as to implement fly control in their barns, stalls, farms, and paddocks. Moreover, horses were recommended to be turned out at certain hours. All of these changes in the management and controlling of insect bites likely lead to decreased exposure and improvement in their underlying problem. This could explain why both the treatment and placebo groups showed clinical improvement without a significant difference between the two. Both groups had better management as this was stressed to the owners at the beginning of the study in order to avoid an additional perpetuating factor.

In addition, our results did not show a significant difference in bacterial counts or inflammatory cell counts from day 0 to day 28. This is unlike what was seen in a previous small animal study on otitis externa in dogs, where a phage cocktail against *P. aeruginosa* resulted in a 30.1% reduction in clinical signs and 67% reduction in *P. aeruginosa* counts after 48 h [21]. 

We postulate that the bacteriophage cocktail could have been capable of killing *S. aureus* as it was designed to, but an overgrowth of commensal or other pathogenic bacteria remained. This could explain the presence of cocci on cytology being similar on day 0 and day 28. This would also explain why, clinically, there was no significant difference in the scores from day 0 to day 28 as other pathogenic cocci were capable of propagating if *S. aureus* was killed and no longer occupying the niche. Therefore, it may be beneficial to create bacteriophage cocktails with multiple different phages as *S. aureus* is not the only pathogenic organism on horses. In the 117 horses that were swabbed at the sites of pyoderma prior to enrollment, only 27.4% contained *S. aureus*, thus showing the importance of other staphylococcal species. Other staphylococcal species that were isolated from the horses with clinical evidence of pyoderma but not enrolled in the study were *S. delphini*, *S. hyicus*, *S. xylosus*, *S. sciuri*, *S. intermedius*, *S. arlettae*, *S. chromogenes*, *S. schleiferi*, *S. succinus*, *S. haemolyticus*, *S. gallinarum*, *S. warneri*, and *S. epidermidis*. The other staphylococcal isolates that were found on the horses at day 28 for both the placebo and treatment groups were *S. chromogenes*, *S. arlettae*, *S. hyicus*, *S. saprophyticus*, *S. succinus*, *S. intermedius*, *S. xylosus*, *S. gallinarium*, *S. dysgalactiae*, *S. delphini*, and *S. hominis*. All of these horses originally had *S. aureus* prior to enrollment. 

When comparing our study with the aforementioned canine otitis externa study, it is possible that the ear study was more successful due to the ears being more of a protected area of the body, allowing bacteriophages to be more efficacious and less vulnerable. The horses in our study were all housed in open farms throughout Florida under strong UV exposure with many weather conditions. The high temperatures, UV rays, and daily topical fly spray application all could have affected the efficacy of the bacteriophage cocktail. This study is also in contrast to human studies that have shown topical bacteriophage to be efficacious. In these human studies, topical bacteriophage was successfully used to treat venous stasis wounds, diabetic foot ulcers, infected burns, and radiation-induced ulcers, among others [33]. In these circumstances, bacteriophages were applied via commercial dressings with the phages impregnated in biodegradable polymer or dripped into the wounds and covered with a phage-soaked gauze so the areas were protected [33]. 

Overall, this pilot study showed that it is possible that topical bacteriophage can kill a specific targeted organism. However, further studies are needed with more controls and a larger sample size. It is difficult to control all the settings on a farm with horses. The underlying cause of many of the pyodermas seen in this study is insect bite hypersensitivity. Therefore, fly sprays are crucial to help prevent further inflammation and infection. Testing phages’ lytic activity with topical fly sprays would be one area of focus that should be addressed. In addition, testing the lytic capability of phages under the harsh weather conditions that animals experience daily would be beneficial. Lastly, it is possible that owner compliance could be of concern and the ideal control would be to have one person perform all applications to ensure standardization. To this end, this study is important for helping investigate novel methods for treating infections. Continual advancements in new options outside of antibiotics is essential for the stewardship of antibiotics and for maximizing the chance of successful therapy. If bacteriophages are going to be the future of treating infections, research into larger cocktails that are able to kill multiple organisms are warranted as this study proved that specific targeting of one main organism is not enough. 

## 5. Conclusions

This study suggests that topical bacteriophage cocktails may be a treatment option in the future for equine pyoderma. Further studies with a larger sample size and greater controls and cocktail formulations are needed to assess and validate the clinical usefulness of this study’s results.

## Figures and Tables

**Table 1:** Scoring table for clinical lesions at weekly visits by the investigator.

	Papules/Pustules	Crusts/Collarettes	Alopecia	Total Score/Body Region
Head/ears				
Neck				
Dorsum				
Cranial ventrum				
Caudal ventrum				
Front legs				
Hind legs				
Treatment site 1				
Treatment site 2				
Total Score				

**Table 2:** Scoring table for clinical lesions at weekly visits by the owner.

	Mild	Moderate	Severe
Site 1			
Site 2			

**Table 3:** Scoring table for cytology at weekly visits.

	Cytology Site 1	Cytology Site 2
Bacterial count		
Inflammatory cell count		

**Table 4:** Phage Susceptibility Test screening against *Staphylococcus aureus* isolates.

	Sa1	Sa2	Sa3	Sa4	Sa5	Sa6	Sa7	Sa8
Sa Phage 1	−	−	−	−	−	−	−	+
Sa Phage 2	−	−	−	−	−	−	−	−
Sa Phage 3	−	−	−	−	−	−	−	−
Sa Phage 4	−	−	−	−	−	−	−	−
Sa Phage 5	−	−	−	−	−	−	−	−
Sa Phage 6	−	−	−	−	−	−	−	−
Sa Phage 7	+	+	−	+	−	+	−	−
Sa Phage 8	+	+	−	−	−	−	−	−
Sa Phage 9	−	+	−	+	−	+	+	+
Sa Phage 10	−	−	−	−	−	−	−	−
Sa Phage 11	−	−	−	−	−	−	−	−
Sa Phage 12	−	−	−	−	−	−	−	−
Sa Phage 13	+	−	+	−	+	+	−	−
Sa Phage 14	+	−	−	−	−	−	−	−
Sa Phage 15	−	+	−	+	−	+	−	−
Sa Phage 16	−	+	−	+	−	+	+	+

*S. aureus* isolates screened against Adaptive Phage Therapeutic’s (APT) PhageBank™ (Gaithersburg, MD, USA) using their Phage Susceptibility Test. The isolates with a positive sign (+) are considered a match for inhibition by a phage, while those with a negative sign (−) do not have inhibition from a phage. Sa, *Staphylococcus aureus*. The phages highlighted in yellow are included in the cocktail.

## Data Availability

No new data were created or analyzed in this study. Data sharing is not applicable to this article.
